# Influence of glide path kinematics during endodontic treatment on the occurrence and intensity of intraoperative and postoperative pain: a systematic review of randomized clinical trials

**DOI:** 10.1186/s12903-020-01164-w

**Published:** 2020-06-22

**Authors:** Thaís Christina Cunha, Felipe de Souza Matos, Luiz Renato Paranhos, Ítalo de Macedo Bernardino, Camilla Christian Gomes Moura

**Affiliations:** 1 Postgraduate Program in Dentistry, School of Dentistry, Federal University of Uberlândia, Uberlândia, MG Brazil; 2 Department of Preventive and Community Dentistry, School of Dentistry, Federal University of Uberlândia, Av. Pará, 1720, Bloco 2G, sala 1, Umuarama, Uberlândia, MG CEP 38405-320 Brazil; 3 Department of Dentistry, School of Dentistry, State University of Paraiba, Campina Grande, PB Brazil; 4 Department of Endodontics, School of Dentistry, Federal University of Uberlândia, Uberlândia, MG Brazil

**Keywords:** Endodontics, Glide path, Pain, Periapical periodontitis, Root canal treatment

## Abstract

**Background:**

Preliminary canal enlargement (glide path preparation) may play a significant role in the development of pain. The aim of this systematic review of randomized clinical trials was to assess the influence of glide path kinematics during endodontic treatment on the occurrence and intensity of intraoperative and postoperative pain.

**Methods:**

A search was performed in June 2019 in six electronic databases (PubMed, Scopus, LILACS, SciELO, Embase and Web of Science) and two grey literature databases (OpenGrey and OpenThesis). The bibliographic references of the eligible articles were also hand-searched. The included clinical studies assessed the occurrence and intensity of intraoperative and/or postoperative pain after root canal preparation without glide path preparation (WGP) or with glide path preparation using manual (M-GP), continuous rotary (CR-GP), or reciprocating (R-GP) instruments. The primary outcome was the occurrence and intensity of intraoperative and postoperative pain, while analgesic consumption was the secondary outcome. The full texts of the eligible studies were analyzed by two reviewers who performed calibration exercises to verify the risk of bias and quality of the individual studies using the Joanna Briggs Institute Critical Appraisal tool.

**Results:**

From 1283 identified articles, only six studies were included in the qualitative analysis of the results, with a total sample of 884 patients/teeth. Three studies presented a high risk of bias, while three studies presented a moderate risk. Two studies reported that CR-GP causes lower pain levels than M-GP and WGP, and three studies showed no differences between CR-GP and R-GP. Regarding analgesic consumption, two studies found no differences among glide path kinematics, and one study reported lower consumption for CR-GP than for M-GP. Because of the limited number of studies and methodological differences, no statistical analyses were performed for the glide path kinematics comparisons.

**Conclusions:**

Compelling evidence indicating a significantly different occurrence and intensity of pain among glide path kinematics is lacking.

The systematic review protocol was registered in the PROSPERO database [CRD42020139989].

## Background

The instrumentation technique used during root canal preparation can play an important role in pain development, as necrotic tissues, contaminated debris and bacteria can be transported to the periapical region, inducing an acute inflammatory response [1–3]. The last decades have been marked by considerable advances in the automated instrumentation of root canals, especially regarding the types of alloy, designs, protocols and kinematics of the files [4].

The use of reciprocating motion was introduced by Yared in 2008 and is an evolution of the balanced force technique [5], which is associated with reduced fatigue fracture of the file, as counterclockwise rotations decrease torsional stresses during the active instrumentation procedure [6, 7]. Despite lower stress values being exerted on the instrument, some studies have linked the performance of this movement to greater debris extrusion compared to continuous rotations [8, 9]; continuous rotations provide a passageway for the removal of debris from the root canal, reducing the incidence of pain and postoperative complications [10]. However, clinical studies on root canal instrumentation comparing reciprocating and continuous rotation kinematics in relation to their effects on debris extrusion and postoperative pain have conflicting results [1, 11–13].

Currently, most automated systems recommend to create a glide path (smooth radicular patency from the root canal orifice to the apical construction) prior to automated root canal instrumentation [14] in order to remove anatomical interferences [15], preserve the original anatomy of the canal [16], and reduce the occurrence of operative accidents, such as canal transportation, step formation, perforations, fractures of nickel-titanium instruments, and apical extrusion of debris [2, 17]. Thus, glide path preparation may also be an important procedure to reduce the occurrence of postoperative pain [18].

Several instruments and techniques have been developed for preinstrumentation, including small diameter stainless steel hand files, such as ISO (International Organization for Standardization) 06, 08 and 10 to ISO 20 [19], and motor-driven nickel-titanium files, which make glide path preparation simpler, safer [20] and faster [21].

Canal transportation and debris extrusion produced by glide path preparation are also considered risk factors that increase postoperative discomfort [22–24]. Thus, the use of stainless steel hand files for glide path preparation, despite their advantages of having a lower cost and better tactile feel than other files [25], tend to rectify the canal path due to their rigidity and create preparations with asymmetrical and irregular wear in all directions of the apical region [23], increasing the incidence of apical canal transportation [25–27]. On the other hand, automated continuous rotation systems such as PathFile® and ProGlider® and reciprocating systems such as R-Pilot® and Wave One Gold Glider® have enhanced modeling capabilities with increased flexibility, and the original geometry of the root canal can thereby be retained [28]. Although previous studies have also reported that manual glide path preparation produces a greater amount of debris extrusion [17, 26], which is associated with the development of pain, the clinical relevance of the relative difference in the incidence of canal transportation and in the amount of apically extruded debris after using stainless steel hand files and continuous rotary or reciprocating glide path systems remains unclear.

Therefore, this study aimed to assess and compare, through a systematic review of randomized clinical trials, the influence of glide path kinematics during endodontic treatment of human permanent teeth on the occurrence and intensity of intraoperative and postoperative pain. The null hypothesis tested was that there is no difference between root canal preparation without glide path preparation (WGP) or with glide path preparation using manual (M-GP), continuous rotary (CR-GP), or reciprocating (R-GP) instruments regarding the occurrence and intensity of intraoperative and postoperative pain.

## Methods

### Protocol and registration

This systematic review was performed according to the recommendations of the PRISMA (Preferred Reporting Items for Systematic Review and Meta-Analysis) statement [29] and the Cochrane guidelines [30]. The systematic review protocol was registered in the PROSPERO database under no. CRD42020139989.

### Study design and eligibility criteria

This study was a systematic review of randomized clinical trials that aimed to answer the following guiding question: "Does the kinematics of glide path preparation during endodontic treatment of human permanent teeth influence the occurrence and intensity of intraoperative or postoperative pain?".

The clinical question under review was structured according to the PICOS format (Population, Intervention, Comparison, Outcome and Study design): P – human permanent teeth, I – glide path preparation, C – alternative glide path preparation or no glide path preparation, O – occurrence and intensity of intraoperative or postoperative pain, S – randomized clinical trials.

The studies included assessed the occurrence and intensity of intraoperative and/or postoperative pain after root canal preparation without glide path preparation (WGP) or with glide path preparation using manual (M-GP), continuous rotary (CR-GP), or reciprocating (R-GP) instruments, without restrictions of the year, language, or publication status (in press).

The following types of articles were excluded: 1) studies not related to the topic and 2) literature reviews, laboratory studies, case reports, case series, letters to the editor or editorials, congress abstracts, personal opinions, and books and/or book chapters.

### Sources of information and search

The PubMed (including MedLine), Scopus, Latin-American and Caribbean Health Sciences Literature (LILACS), SciELO, Embase and Web of Science databases were the primary sources that were searched. OpenGrey and OpenThesis partially covered the “grey literature”. A manual search was also performed through a systematized analysis of the references of the eligible articles. All steps were performed with the aim of minimizing selection and publication biases.

MeSH (Medical Subject Headings), DeCS (Health Sciences Descriptors), and Emtree (Embase Subject Headings) were used to select the search descriptors. Several combinations of the Boolean operators “AND” and “OR” enhanced the research strategy (Table 1). The bibliographic research was performed in June 2019. The results obtained were exported to EndNote Web™ software (Thomson Reuters, Toronto, Canada), with which duplicates were removed. The remaining results were exported to Microsoft Word™ 2010 (Microsoft™ Ltd, Washington, USA), with which the remaining duplicates were manually removed.

**Table 1:** Strategies for database search

Database	Search Strategy (June, 2019)
PubMed	((“Apical Periodontitis” OR “Dental Pulp Disease” OR “Dental Pulp Necrosis” OR “Periapical Disease” OR “Periapical Periodontitides” OR “Pulpitis” OR “Root Canal” OR “Root Canals” OR “Teeth” OR “Tooth”) AND (“Glide Path” OR “Hyflex GPF” OR “Nickel-Titanium Rotary Instruments” OR “One G” OR “PathFile” OR “ProGlider” OR “R-Pilot” OR “Scout RaCe” OR “Wave One Gold Glider”))
http://www.ncbi.nlm.nih.gov/pubmed	
Scopus	((“Apical Periodontitis” OR “Dental Pulp Disease” OR “Dental Pulp Necrosis” OR “Periapical Disease” OR “Periapical Periodontitides” OR “Pulpitis” OR “Root Canal” OR “Root Canals” OR “Teeth” OR “Tooth”) AND (“Glide Path” OR “Hyflex GPF” OR “Nickel-Titanium Rotary Instruments” OR “One G” OR “PathFile” OR “ProGlider” OR “R-Pilot” OR “Scout RaCe” OR “Wave One Gold Glider”))
http://www.scopus.com/	
LILACS	(“Glide Path” OR “Nickel-Titanium Rotary Instruments” OR “PathFile”)
http://lilacs.bvsalud.org/	
SciELO	(“Glide Path” OR “Nickel-Titanium Rotary Instruments” OR “PathFile”)
http://www.scielo.org/	
Embase	(‘apical periodontitis’/exp OR ‘apical periodontitis’ OR ‘dental pulp disease’/exp OR ‘dental pulp disease’ OR ‘dental pulp necrosis’/exp OR ‘dental pulp necrosis’ OR ‘periapical disease’/exp OR ‘periapical disease’ OR ‘periapical periodontitides’ OR ‘pulpitis’/exp OR ‘pulpitis’ OR ‘root canal’/exp OR ‘root canal’ OR ‘root canals’ OR ‘teeth’/exp OR ‘teeth’ OR ‘tooth’/exp OR ‘tooth’) AND (‘glide path’ OR ‘hyflex gpf’ OR ‘nickel-titanium rotary instruments’ OR ‘one g’ OR ‘pathfile’ OR ‘proglider’ OR ‘r-pilot’ OR ‘scout race’ OR ‘wave one gold glider’)
http://www.embase.com	
Web of Science	((“Apical Periodontitis” OR “Dental Pulp Disease” OR “Dental Pulp Necrosis” OR “Periapical Disease” OR “Periapical Periodontitides” OR “Pulpitis” OR “Root Canal” OR “Root Canals” OR “Teeth” OR “Tooth”) AND (“Glide Path” OR “Hyflex GPF” OR “Nickel-Titanium Rotary Instruments” OR “One G” OR “PathFile” OR “ProGlider” OR “R-Pilot” OR “Scout RaCe” OR “Wave One Gold Glider”))
http://apps.webofknowledge.com/	
OpenGrey	(“Glide Path” OR “Nickel-Titanium Rotary Instruments” OR “PathFile”)
http://www.opengrey.eu/	
OpenThesis	(Glide Path OR Nickel-Titanium Rotary Instruments OR PathFile)
http://www.openthesis.org/	

### Study selection

Before starting the study selection process, as a calibration exercise, the reviewers discussed the eligibility criteria and applied them to a sample of 20% of the studies retrieved to determine interexaminer agreement. After achieving a proper level of agreement (Kappa ≥ 0.81), the study selection process was performed in three different phases by two eligibility reviewers. In the first phase, the reviewers independently performed a methodical analysis of the titles of the studies. The reviewers were not blinded to the names of authors and journals. Articles with titles not related to the topic were eliminated in this phase. In phase 2, two reviewers also analyzed the abstracts systematically and excluded studies according to the eligibility criteria. The studies that were related to the topic but did not have abstracts available were fully analyzed in the third phase. In this phase, the full texts of the preliminary eligible studies were analyzed to verify whether they fulfilled the eligibility criteria. When an agreement could not be reached in the assessment, a third reviewer was consulted to make a final decision.

### Process of data collection and extraction

After selection, the studies were analyzed, and two reviewers extracted data on the following information: identification of the study (author, year of publication, and study location); sample characteristics (number of patients or teeth, distribution by sex, mean age and range, types of teeth, inclusion criteria, study groups, and outcome measure); and intervention characteristics (operators, working length, glide path system, instrumentation system, surgical diameter, number of sessions, and root canal filling, if applicable).

To ensure consistency among reviewers, both reviewers performed a calibration exercise during which information was extracted jointly from an eligible study. The reviewers solved any disagreements through discussions, and when both reviewers disagreed, they consulted a third reviewer for a final decision.

### Risk of bias and individual quality of the studies

The risk of bias and individual quality of the studies selected was assessed by the Joanna Briggs Institute Critical Appraisal Tools for use in JBI Systematic Reviews Checklist for Randomized Controlled Trials [31]. Two authors assessed each domain independently and systematically regarding the potential risk of bias, as recommended by the PRISMA [29]. The reviewers solved any disagreements through discussions, and when both reviewers disagreed, they consulted a third reviewer for a final decision.

The following criteria were used for the assessment: Q.1) Was true randomization used for assignment of participants to treatment groups?; Q.2) Was allocation to treatment groups concealed?; Q.3) Were treatment groups similar at the baseline?; Q.4) Were participants blind to treatment assignment?; Q.5) Were those delivering treatment blind to treatment assignment?; Q.6) Were outcomes assessors blind to treatment assignment?; Q.7) Were treatment groups treated identically other than the intervention of interest?; Q.8) Was follow up complete and if not, were differences between groups in terms of their follow up adequately described and analyzed?; Q.9) Were participants analyzed in the groups to which they were randomized?; Q.10) Were outcomes measured in the same way for treatment groups?; Q.11) Were outcomes measured in a reliable way?; Q.12) Was appropriate statistical analysis used?; Q.13) Was the trial design appropriate, and any deviations from the standard RCT design (individual randomization, parallel groups) accounted for in the conduct and analysis of the trial?.

The risk of bias for a study was ranked as “high” when up to 49% of the answers corresponded to "yes", “moderate” when 50% to 69% of the answers corresponded to "yes", and “low” when more than 70% of the answers corresponded to "yes".

### Summary measures

The occurrence and intensity of intraoperative and postoperative pain after glide path preparation were the main outcomes evaluated. Analgesic consumption, i.e., the number of analgesic tablets consumed for pain resolution, was the secondary outcome. Intraoperative pain assessments included assessments of pain performed during the first treatment session and immediately after glide path preparation and/or root canal instrumentation. Postoperative pain assessments included those performed after the end of the first treatment session and between 6 and 72 hours after obturation and/or temporary filling of the crown.

## Results

### Study selection

**Fig. 1:**
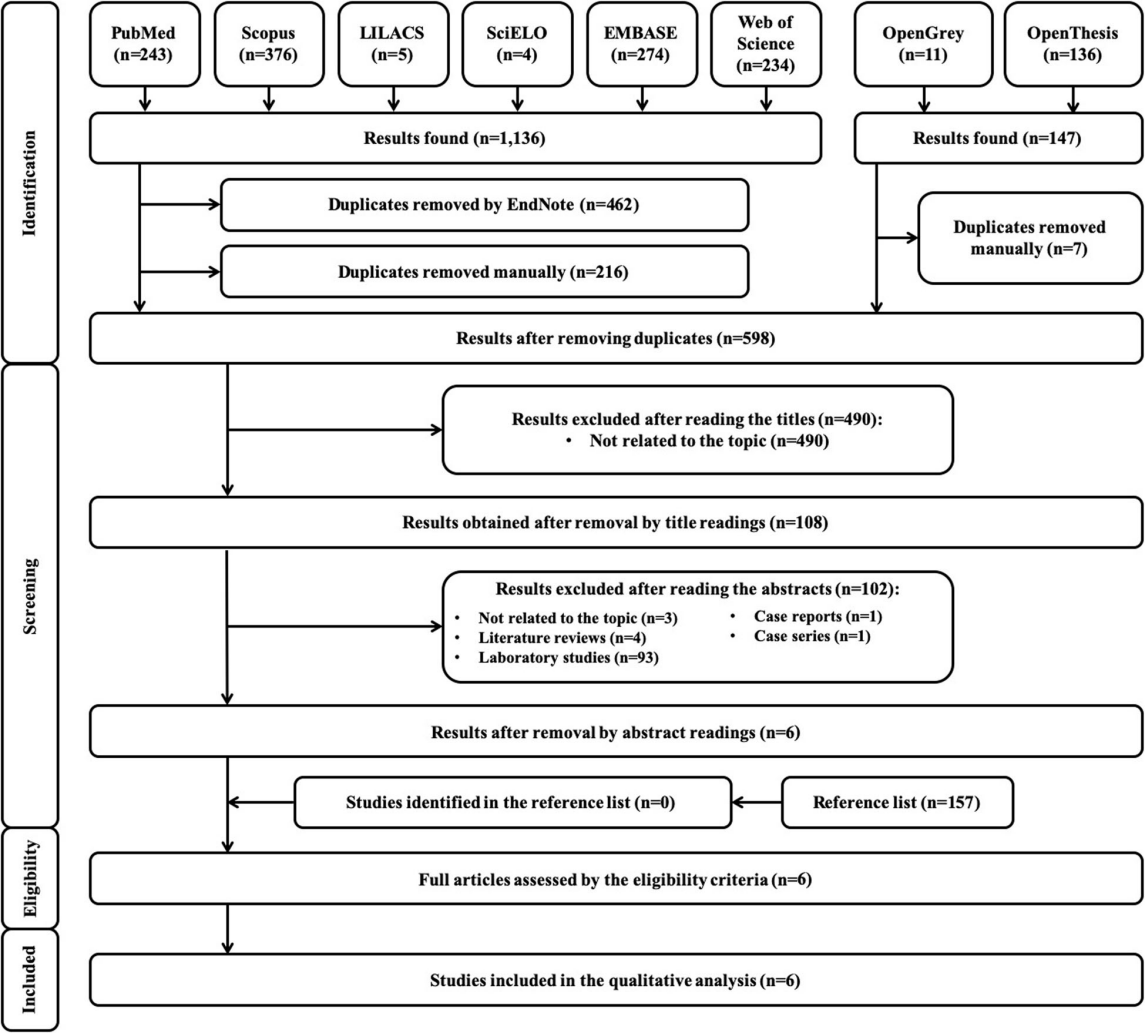
Flowchart adapted from the PRISMA statement showing the literature search and selection processes.

The first selection phase resulted in 1283 studies distributed in eight electronic databases. After the duplicates were removed, 598 studies remained for the analysis of the titles and abstracts. Then, after the titles were read, 108 studies remained for the analysis of the abstracts. After the abstracts were analyzed, only six studies were considered eligible for the full text assessment. The references of the six studies were carefully assessed to identify studies retrieved through the main search strategy, but none were found. Therefore, the six studies [2, 4, 18, 32–34] were included in the qualitative analysis of the results. Figure 1 shows the search, identification, inclusion, and exclusion processes for the articles.

### Study characteristics

Table 2 shows a summary of the main population characteristics of the studies. The analysis of the six studies resulted in a total sample of 884 patients/teeth, whose ages ranged from 16 to 70 years. From the six studies analyzed, only three [2, 4, 34] mentioned the ethical criteria involved, three [2, 4, 34] followed the CONSORT statement, and two reported the use of informed consent forms for the research participants [4, 34]. Table 3 shows a summary of the main intervention characteristics of the studies. Pain occurrence and intensity were evaluated after several operative steps: during the operative session, after glide path preparation [34] or after both glide path preparation and root canal instrumentation [32, 33]; following the first treatment session, after glide preparation, root canal instrumentation and obturation with gutta-percha and AH Plus sealer [2, 4], or after glide path preparation and the placement of a cotton pellet in the root canals and temporary filling [18].

**Table 2:** Summary of the main population characteristics of the eligible studies

Author, year, and country	Sample (n) and sex	Mean sample age and range (years)	Types of teeth	Inclusion criteria	Study groups	Outcome measure
Pasqualini et al., 2012. Italy [18].	295 patients (+)	42 (16–70)	Single rooted and multirooted teeth	Asymptomatic irreversible pulpitis, symptomatic irreversible pulpitis, or pulp necrosis with or without apical periodontitis	^I^CR-GP (PathFile) and^II^M-GP (Stainless-steel K-file)	Postoperative pain
						Analgesic consumption
Chen et al., 2013. China [32].	88 teeth (+)	+	Molars and premolars	Acute or chronic pulpitis or periapical periodontitis	^I^CR-GP (PathFile + Reciproc), ^II^CR-GP (PathFile + ProTaper), and ^III^WGP (ProTaper)	Intraoperative pain
Guo et al., 2014. China [33].	80 patients (+)	+ (17–60)	First and second molars	Pulp inflammatory disease	^I^CR-GP (PathFile, experienced physicians), ^II^CR-GP (PathFile, unexperienced physicians), ^III^M-GP (Stainless-steel K-file, experienced physicians), and ^IV^M-GP (Stainless-steel K-file, unexperienced physicians)	Intraoperative pain
Adıgüzel et al., 2019. Turkey [2].	93 patients (43♀ 50♂)	40 (20–65)	Single-canaled mandibular premolars	Asymptomatic non-vital pulp	^I^CR-GP (One G), ^II^R-GP (R-Pilot) and ^III^WGP	Postoperative pain
						Analgesic consumption
Keskin et al., 2019. Turkey [4].	240 patients (137♀ 103♂)	+ (18–60)	Maxillary and mandibular teeth	Asymptomatic irreversible pulpitis, symptomatic irreversible pulpitis, symptomatic apical periodontitis or asymptomatic apical periodontitis	^I^R-GP (R-Pilot), ^II^CR-GP (ProGlider) and ^III^M-GP (stainless-steel K-file)	Postoperative pain
						Analgesic consumption
Tüfenkçi et al., 2019. Turkey [34].	88 patients (50♀ 38♂)	40 (18–69)	First and second mandibular molars	Asymptomatic irreversible pulpitis	^I^R-GP (R-Pilot), ^II^R-GP (WaveOne Gold Glider), ^III^CR-GP (One G) and ^IV^CR-GP (ProGlider)	Intraoperative pain

Superscript roman numerals mean group number; +Not mentioned by the author; ♀ Women; ♂ Men; *M-GP* manual glide path, *CR-GP* continuous rotary glide path, *R-GP* reciprocating glide path, *WGP* without glide path.

**Table 3:** Summary of the main intervention characteristics of the eligible studies

Authors	Operators	Working length	Glide path system	Instrumentation system	Surgical diameter	No of sessions	Root canal filling
Pasqualini et al., 2012 [18]	21 endodontists	Full root canal length, i.e. up to the apical foramen	^I^CR-GP (PathFile #13, #16, #19, taper 0.02) and ^II^M-GP (Stainless-steel K-file #08, #10, #12, #15, #17, #20)	#	#	1	Empty (cotton pellet)
Chen et al., 2013 [32]	Single operator	Full root canal length, i.e. up to the apical foramen	^I,II^CR-GP (PathFile #13, #16, #19, taper 0.02) and ^III^WGP	^I^Reciproc (R25), ^II,III^ProTaper (#S1, #S2, #F1, #F2)	25/0.08	#	#
Guo et al., 2014 [33]	Experienced^I,III^ or unexperienced^II,IV^ physicians	Full root canal length, i.e. up to the apical foramen	^I,II^CR-GP (PathFile #13, #16, #19, taper 0.02) and ^III,IV^M-GP (Stainless-steel K-file #10, #15, #20)	ProTaper	+	#	#
Adıgüzel et al., 2019 [2]	Single endodontist	Full root canal length, i.e. up to the apical foramen	^I^CR-GP (One G #14, taper 0.03), ^II^R-GP (R-Pilot #12.5, taper 0.04) and ^III^WGP	Mtwo	30/0.05	1	Gutta-percha and AH Plus sealer
Keskin et al., 2019 [4]	Four endodontists	Full root canal length, i.e. up to the apical foramen	^I^R-GP (R-Pilot #12.5, taper 0.04), ^II^CR-GP (ProGlider #16, variable taper), and ^III^M-GP (stainless-steel K-file #08, #10, #15)	ProTaper Next	30/0.07, 40/0.06 or 50/0.06	1	Gutta-percha and AH Plus sealer
Tüfenkçi et al., 2019 [34]	Single operator	Full root canal length, i.e. up to the apical foramen	^I^R-GP (R-Pilot #12.5, taper 0.04), ^II^R-GP (WaveOne Gold Glider #17, variable taper), ^III^CR-GP (One G #14, taper 0.03), ^IV^CR-GP (ProGlider #16, variable taper)	#	#	#	#

Superscript roman numerals mean group number; +Not mentioned by the author; #Not applicable; *M-GP* manual glide path, *CR-GP* continuous rotary glide path, *R-GP* reciprocating glide path, *WGP* without glide path.

### Risk of bias and individual quality of the studies

Table 4 shows information regarding the risk of bias and individual quality of the studies included in this systematic review. According to the analysis of the JBI Critical Appraisal Checklist for Randomized Controlled Trials [31], three studies [18, 32, 33] presented high risk of bias, while three studies [2, 4, 34] presented moderate risk. Questions Q.1 and Q.2 were considered “unclear” for three studies [18, 32, 33] because the authors stated that the patients were randomly allocated to the study groups, but the authors did not provide details about the randomization procedure and allocation concealment, respectively. Question Q.5 was considered “not applicable” for all studies [2, 4, 18, 32–34] because the operator could not be blind to the instrumentation technique he/she is using. Questions Q.6 and Q.11 were considered “not applicable” for all studies [2, 4, 18, 32–34] because the results were evaluated by means of a pain scale form or visual analogue scale completed by the research participants themselves. Question Q.9 was considered “not applicable” for three studies [2, 33, 34] because there was no loss of patients included in the trial.

**Table 4:** Risk of bias and individual quality of the studies assessed by the Joanna Briggs Institute Critical Appraisal Tools for use in JBI Systematic Reviews for Randomized Controlled Trials. The risk of bias was classified as high when the study reached up to 49% of "yes" score, moderate when the study reached from 50% to 69% of "yes" score, and low when the study reached more than 70% of "yes" score

Authors	Q.1	Q.2	Q.3	Q.4	Q.5	Q.6	Q.7	Q.8	Q.9	Q.10	Q.11	Q.12	Q.13	% yes/risk
Pasqualini et al., 2012 [18]	U	U	√	--	N/A	N/A	√	√	--	√	N/A	√	√	46% yes/ high risk of bias
Chen et al., 2013 [32]	U	U	√	--	N/A	N/A	√	--	--	√	N/A	√	√	38% yes/ high risk of bias
Guo et al., 2014 [33]	U	U	√	--	N/A	N/A	√	√	N/A	√	N/A	√	√	46% yes/ high risk of bias
Adıgüzel et al., 2019 [2]	√	√	√	--	N/A	N/A	√	√	N/A	√	N/A	√	√	61% yes/ moderate risk of bias
Keskin et al., 2019 [4]	√	√	√	--	N/A	N/A	√	√	--	√	N/A	√	√	61% yes/ moderate risk of bias
Tüfenkçi et al., 2019 [34]	√	√	√	√	N/A	N/A	√	√	N/A	√	N/A	√	√	69% yes/ moderate risk of bias

√ - Yes; -- - No; *U* Unclear, *N/A* Not applicable.

### Specific results of the eligible studies

**Fig. 2:**
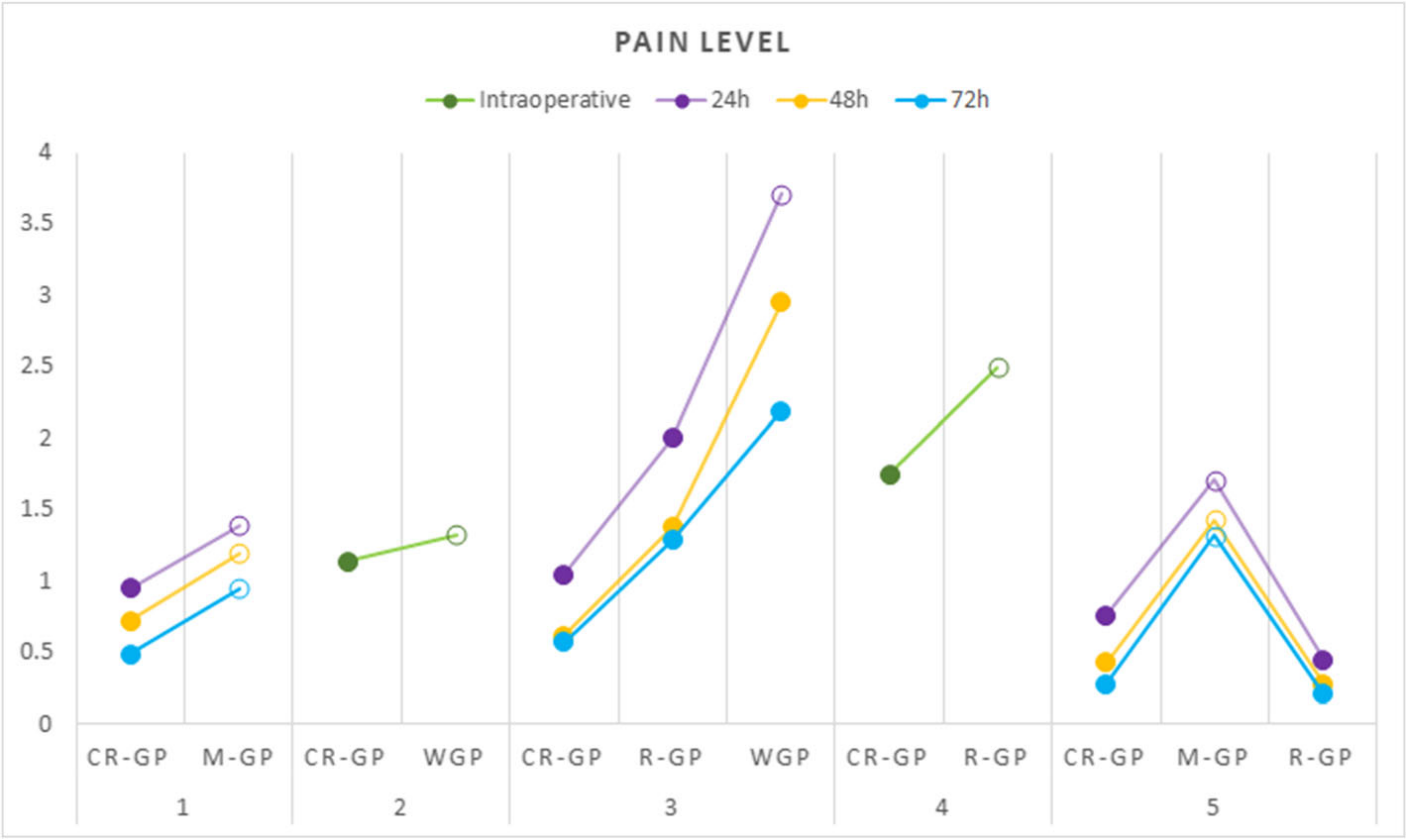
Mean values of the intensity of intraoperative and postoperative pain reported in the eligible studies after glide path preparation with different kinematics: M-GP, manual glide path; CR-GP, continuous rotary glide path; R-GP, reciprocating glide path; WGP, without glide path. 1, Pasqualini et al. [18]; 2, Chen et al. [32]; 3, Adiguzel et al. [2]; 4, Tufenkçi et al. [34]; 5, Kesklin et al. [4]. Unfilled circles represent a significantly higher level of pain than the other groups in the same assessment period and in each study. In the study by Tufenkçi et al. [34], the only one in which pain level values were expressed as medians, the intensity of intraoperative pain attributed to R-GP was obtained by calculating the mean of R-GP (RP) and R-GP (WOGG) medians, and the intensity of intraoperative pain attributed to CR-GP was obtained by calculating the mean of CR-GP (OP) and CR-GP (PG) medians. The study by Guo et al. [33] was not included in the graph because it did not report intraoperative pain levels in the studied groups, but only the percentage of pain occurrence: 15-20% for CR-GP and 25-35% for MG-GP, with a statistically significant difference between them.

Table 5 shows a summary of the parameters and results collected for the studies included in the qualitative analysis. Three studies [32–34] evaluated the occurrence and intensity of intraoperative pain, and three studies [2, 4, 18] evaluated the occurrence and intensity of postoperative pain and analgesic consumption. In Table 5, for the comparison between postoperative pain levels in each study, only the 24-, 48- and 72-hour periods were considered because these were the periods evaluated in the three studies [2, 4, 18]. Figure 2 shows the intensity of intraoperative and postoperative pain reported in the eligible studies after glide path preparation with different kinematics. Considering the individual results of the eligible studies concerning the influence of glide kinematics on the occurrence and intensity of intraoperative and postoperative pain, the following statements can be made: 1) Two studies [4, 18] reported that CR-GP leads to lower pain levels than M-GP, but one study [33] found no difference between CR-GP and M-GP, and two studies [2, 32] showed better results for CR-GP than for WGP. 2) One study [4] reported that R-GP leads to lower pain levels than M-GP, but another study [2] found no difference between R-GP and WGP. 3) Three studies [2, 4, 34] showed no differences between CR-GP and R-GP, and one study [34] reported that CR-GP causes lower pain levels than R-GP. Regarding analgesic consumption, two studies [2, 4] found no differences among M-GP, CR-GP, R-GP and WGP, and only one study [18] reported lower analgesic consumption per subject for CR-GP than for M-GP.

**Table 5:** Summary of the parameters and results collected for the studies included in the qualitative analysis

	Intraoperative and postoperative pain assessment				Analgesic consumption (mean ± SD)
Authors	Method	Period	Classification	Results (mean ± SD)	
Pasqualini et al., 2012 [18]	5-level pain scale form	24, 48, 72 h	No pain (0), slight pain (1), mild pain (2), severe pain (3), very severe pain (4), extremely severe pain (5)	24 h: ^II^M-GP (1.38) > ^I^CR-GP (0.94)	^II^M-GP (3.7 ± 2.2) > ^I^CR-GP (2 ± 1.7)
				48 h: ^II^M-GP (1.19) > ^I^CR-GP (0.71)	
				72 h: ^II^M-GP (0.95) > ^I^CR-GP (0.48)	
Chen et al., 2013 [32]	3-level pain scale form	#	Completely painless (1); mild pain, does not affect occlusion and eating (2); severe pain, affecting occlusion and eating (3)	^III^WGP (1.33 ± 0.55) > ^I,II^CR-GP (1.14 ± 0.36)	#
Guo et al., 2014 [33]	Visual analogue scale	#	10 cm ruler marked 0 to 10 scale: pain (value 3-10) or no pain (value <3)	^I,II^CR-GP (15-20%) = ^III,IV^M-GP (25-35%)	#
Adıgüzel et al., 2019 [2]	Visual analogue scale	24, 48, 72 h	No pain (0), mild pain (1–3), moderate pain (4–6), severe pain (7–10)	24 h: ^II^R-GP (2.00 ± 1.87) = ^III^WGP (3.71 ± 2.03) > ^I^CR-GP (1.05 ± 1.07) = ^II^R-GP	^I^CR-GP (0.61 ± 0.95) = ^II^R-GP (0.74 ± 0.96) = ^III^WGP (1.06 ± 1.06)
				48 h: ^I^CR-GP (0.62 ± 0.67) = ^II^R-GP (1.38 ± 0.80) = ^III^WGP (2.95 ± 1.36)	
				72 h: ^I^CR-GP (0.57 ± 0.68) = ^II^R-GP (1.29 ± 1.06) = ^III^WGP (2.19 ± 1.33)	
Keskin et al., 2019 [4]	Visual analogue scale	24, 48, 72 h	+	24 h: ^III^M-GP (1.71) > ^I^R-GP (0.45) = ^II^CR-GP (0.76)	^I^R-GP (0.0 ± 0.0) = ^II^CR-GP (0.0 ± 0.0) = ^III^M-GP (0.0 ± 0.0)
				48 h: ^III^M-GP (1.43) > ^I^R-GP (0.28) = ^II^CR-GP (0.43)	
				72 h: ^III^M-GP (1.32) > ^I^R-GP (0.21) = ^II^CR-GP (0.28)	
Tüfenkçi et al., 2019 [34]	Visual analogue scale	#	No pain (0), mild pain (1–3), moderate pain (4–6), severe pain (7–10)	^III^CR-GP (2 ± 0.63*) = ^I^R-GP (2 ± 0.95*) = ^II^R-GP (3 ± 1.01*) > ^IV^CR-GP (1.5 ± 0.80*) = ^III^CR-GP	#

Superscript roman numerals mean number of groups; +Not mentioned by the author; #Not applicable; >statistically significant difference; =not statistically significant difference; *Data expressed as median ± SD; *M-GP* manual glide path, *CR-GP* continuous rotary glide path, *R-GP* reciprocating glide path, *WGP* without glide path.

## Discussion

Preliminary manual or mechanical root canal enlargement (glide path preparation) has been recommended to reduce the anatomical interferences of the canal walls and to soften the descent path of the instruments used in the chemical-mechanical preparation [15]. This procedure facilitates root canal preparation, enabling a safer use of files and thus preventing instrument fractures [16, 35], root canal deformation [18, 23, 36] and apical extrusion of contaminated debris [2, 17]. Different glide path systems with variable kinematics are commercially available, and their effects on apical debris extrusion and influence on the development of postoperative symptoms remain unclear. Therefore, the objective of this systematic review was to search the available literature for clinical evidence supporting a relationship between glide path kinematics and intra- and postoperative symptoms to guide the selection of a glide path system for safe clinical use and to improve patients’ well-being, thus improving the treatment prognosis.

The null hypothesis was rejected since glide path kinematics during endodontic treatment may influence the development of pain. However, the moderate [2, 4, 34] or high [18, 32, 33] risk of bias as well as the heterogeneity of the included studies made direct comparisons among them or statistical manipulations such as a meta-analysis impossible, decreasing the evidence strength of the results of this systematic review. This issue draws the attention of the scientific community to the need for the standardization of clinical study designs according to the CONSORT guidelines, with which important information should be evaluated before starting the research study and before the publication of the results. Allocation concealment, blinding the participants, operators and outcome assessors to the treatment assignments are important factors in minimizing confounders and avoiding performance bias [37]. Although all clinical trials included in this systematic review used randomization [2, 4, 18, 32–34], three of them [18, 32, 33] did not describe the randomization process and did not provide details on the allocation concealment method used for assigning the participants to treatment groups. In five works [2, 4, 18, 32, 33], the people involved were not blinded.

One of the main concerns regarding the study of pain is its form of assessment since pain is a sensory, subjective and intrapersonal experience whose threshold varies between subjects [18, 38]. According to *Conti* et al., no “standard” is available to quantify pain, and the visual analogue scale (VAS) is the most suitable method for pain assessment [39]. VAS corresponds to a horizontal line of 100 mm, without numbers, whose minimum reading refers to “no pain” and the maximum reading to “the worst imaginable pain” by the patient. This scale is a simple and important tool to assess the prevalence and severity of pain and changes resulting from treatments [39]. In addition, VAS is considered accurate because it has few steps and is reliable because the patient would not be biased by numbers usually present in standard numerical scales that are not very objective due to the heterogeneity of personal character [39, 40]. In the included studies, pain was assessed primarily through a visual analogue scale (VAS) [2, 4, 33, 34], but two studies applied numbered scales [18, 32]. In addition, there were differences in the definitions of the pain scales used, that is, the same degree of pain may have different meanings in different scales, which make it impossible to directly compare the results of the studies or statistical combinations of the results. Although the VAS is a simple and valuable tool for assessing pain intensity and alterations due to treatment, it does not allow the cause of pain to be determined, making the clinical significance of the VAS scores questionable [38, 41]. The presence of preoperative pain as reported in four studies [4, 18, 32, 33] reinforces this argument, as it is considered the major determinant or prognostic factor of postoperative pain [4, 42, 43]. Thus, pain intensity should be reported in relation to its improvement or deterioration, with a decrease or increase in a numerical value, regardless of the scale used, to make the results clinically important and comparable [44].

Pain perception is characterized by inter-individual variability related to sex and age, their level of catastrophizing and anxiety [45, 46]. In this systematic review, three studies evaluated pain in both male and female patients, with homogeneous distributions [2, 4, 34]. However, it has been reported that females typically tend to display lower pain thresholds and pain tolerance than men [47, 48]. Many studies have sought to clarify the female predominance in the prevalence of pain, and it is believed that females suffer more commonly from psychosomatic illnesses and that their pain is governed by emotional factors [49]. More legitimate explanations are based on biological variations between the sexes: differences in pelvic levels and reproductive organs; hormonal factors associated with changes in serotonin and norepinephrine levels, leading to an increased prevalence of pain during menstrual periods [50, 51] and hormone replacement processes or the use of oral contraceptives [52]. Several hypotheses have also been proposed to explain the relationship between age and pain. The selected articles evaluated patients ranging in age from 16 to 70 years [2, 4, 18, 33, 34]. The literature reports that with aging, there is an increase in the pain threshold in response to stimuli. Because pain is a function of the brain, it is subject to age-related changes, so interactions between brain aging and pain processing are likely to interfere with the experience of pain in older people. Studies have suggested peripheral changes, such as impaired activity of delta A myelinated fibers and structural changes in specific regions of the central nervous system, which is involved in pain processing, to be possible reasons for changes in the perception of pain [53–55]. These findings suggest that in future studies, pain should be measured in relation to sex and age to reduce bias in pain assessments and treatments, thus leading to a better understanding of the factors that influence pain perception during or after endodontic treatment.

Although all root canal instrumentation techniques are associated with debris extrusion, the amount of debris extruded may vary depending on the instrument’s kinematics and design [2, 3, 56, 57]. When transported to periapical tissues, infected debris can induce an acute or chronic inflammatory response with or without pain and swelling [18, 48, 56, 58]. Considering the individual results of the studies included in this systematic review, CR-GP or R-GP may cause lower rates of intraoperative and postoperative pain compared to root canal preparation without glide path (WGP) or with M-GP (Fig. 2). Other studies have also shown that most motor-driven nickel titanium (NiTi) instruments extrude less debris into the periapical tissues than manual instruments due to their rotary kinematics associated with abundant irrigation, minimizing postoperative discomfort [57, 59–61]. In addition, both rotary and reciprocating kinematics have shown to exert similar and minimal effects on the intraoperative discomfort of patients [62]. However, in the study by *Hou* et al., the incidence of postoperative pain was lower in patients treated with rotary instruments than in those treated with reciprocating instruments because there was less debris extrusion, which reduces irritation and minimizes inflammation and the release of chemical mediators, such as neuropeptides, arachidonic acid metabolites, cytokines, lysosomal enzymes, platelet activation factors, fibrinolytic peptides, vasoactive amines, anaphylatoxins and kinins, which can lead to postoperative complications [10, 63].

Other intraoperative factors can also influence the development of pain including the number of treatment sessions, the type of chemical substance used in the root canal instrumentation and intracanal medication, as well as the root canal obturation technique [48, 61]. Several irrigants are used during endodontic therapy, with sodium hypochlorite (NaOCl) being the most commonly used due to its antimicrobial and antibiofilm activities, and the dissolving power of organic tissues [64, 65]. On the other hand, when extruded to the periapex, it is irritating to periapical tissues, especially in high concentrations (5.25%), and even in low concentrations (0.5%) it can induce an inflammatory reaction, which increases the probability of occurrence of intra and postoperative pain [64–66]. Standardizing irrigation protocols, such as controlling the depth of needle penetration, can help eliminate the effects of intraoperative variables on results [67]. Four of the selected articles [2, 4, 18, 32] reported the use of 5.25% hypochlorite as an irrigating solution, but none of them provided information about the irrigation technique and control of irrigating solution extrusion, making it impossible to define the interference of substances used during the endodontic treatment in the incidence of pain.

In two studies [2, 4], analgesic consumption was not influenced by glide path kinematics, whereas in the study by *Pasqualini* et al. analgesic consumption was reduced in the patients treated with continuous rotary glide path preparation compared with those who underwent manual glide path preparation [18]. Severe postoperative pain requiring posttreatment analgesic medication is commonly associated with biomechanical preparation procedures that stimulate an immune response to the irrigants and microorganisms present in extruded debris, overinstrumentation, or foreign body reactions to filling materials [68]. In the study by *Pasqualini* et al., the manual glide path procedure probably increased analgesic consumption compared with the continuous rotary glide path procedure by extruding more contaminated debris into the periapex [17, 18]. Pulpal and periodontal diagnoses, pre-operative pain and intraoperative factors such as irrigation solution and system, instrumentation technique, use and type of medicament, root canal filling technique and occlusal reduction have been previously shown to influence post-operative pain and could justify differences between study results [3, 69–72].

The main limitation of this systematic review is the low number of eligible articles that may compromise the strength of the evidence. In addition, methodological differences related to sample and intervention characteristics limited the evaluation of results and direct comparisons of the investigated outcomes among the eligible articles. Thus, some divergences in clinical findings may have been caused by the variation in the instrumentation protocols, the number and clinical experience of operators [73, 74] and the systems used [12, 24]. However, this systematic review answers a relevant and unpublished clinical question, thereby contributing to an improvement in glide path protocols during endodontic treatment, its prognosis, and therapeutic success levels. Additional randomized controlled clinical trials that avoid the aforementioned limitations should be conducted in accordance with the CONSORT guidelines to provide more compelling evidence on the influence of glide path kinematics on pain development.

## Conclusion

Considering the individual results of the eligible studies, glide path kinematics during endodontic treatment may influence the development of pain. Continuous rotary (CR-GP) or reciprocating (R-GP) glide path systems appear to cause lower rates of intraoperative and postoperative pain compared to root canal preparation without glide path preparation (WGP) or with glide path using manual instruments (M-GP). However, compelling evidence indicating a significantly different occurrence and intensity of pain among glide path kinematics is lacking. Variability in treatment protocols, patient selection, and treatment effects made it impossible to statistically compare the individual results of studies.

## Data Availability

The datasets of this article are available from the corresponding author on reasonable request.

## Abbreviations

WGP: Without Glide Path
M-GP: Manual Glide Path
CR-GP: Continuous Rotary Glide Path
R-GP: Reciprocating Glide Path
ISO: International Organization for Standardization
PRISMA: Preferred Reporting Items for Systematic Review and Meta-Analysis
JBI: Joanna Briggs Institute
VAS: Visual Analogue Scale
